# Genome Sequence of *Arthrobacter* sp. YC-RL1, an Aromatic Compound-Degrading Bacterium

**DOI:** 10.1128/genomeA.00749-15

**Published:** 2015-07-09

**Authors:** Lei Ren, Yanhua Shi, Yang Jia, Yanchun Yan

**Affiliations:** Graduate School of Chinese Academy of Agricultural Sciences, Beijing, China

## Abstract

We report the 4.04-Mb draft genome sequence of *Arthrobacter* sp. YC-RL1, an aromatic compound-degrading bacterium. YC-RL1 could degrade a wide range of aromatic compounds, including naphthaline, 1,2,3,4-tetrachlorobenzene, fluorene, 4-nitrophenol, bisphenol A, biphenyl, and *p*-xylene. The genome sequence of YC-RL1 will promote the investigation of the biodegradation of aromatic compounds.

## GENOME ANNOUNCEMENT

The genus *Arthrobacter* is widely distributed in the environment and has been found to be involved in the metabolism of xenobiotics (1, 2). Several strains of *Arthrobacter* spp. have been found to be capable of degrading aromatic compounds, including *Arthrobacter phenanthrenivorans* Sphe3, *Arthrobacter* sp. W1, *Arthrobacter chlorophenolicus* A6, and *Arthrobacter* sp. SJCon (3–6). *Arthrobacter* sp. strain YC-RL1 could utilize a wide range of aromatic compounds as sole carbon sources for growth. Aromatic compounds including naphthalene, fluorine, 4-nitrophenol, 1,2,3,4-tetrachlorobenzene, bisphenol A, biphenyl, and *p*-xylene were tested. The degradation rate of the substrates mentioned by strain YC-RL1 were all above 90% after a 7-day incubation (different substrate was served as sole carbon source separately). Compared with other reported *Arthrobacter* species, strain YC-RL1 has a wider substrate range. Thus, the genome report of YC-RL1 may provide more comprehensive genetic information for the understanding of xenobiotic metabolism and the application of this strain in environmental bioremediation in the future.

The genome of strain YC-RL1 was sequenced using the Illumina HiSeq 2500 platform with a 600-bp paired-end library and a 3-kb mate pair library. A total of 1,595,680,964 bp of original data were generated (approximately 325× coverage of the genome), with an average G+C content of 64.01%. The original data were assembled into 17 large contigs using Velvet version 1/2/10 software, with a calculated 4,037,349-bp total length and an *N*_50_ contig size of 2,650,074 bp. Tandem repeats were identified by Tandem Repeat Finder version 4.02 (http://tandem.bu.edu/trf/trf.html) (7). The genome was functionally annotated through the NCBI Prokaryotic Genome Automatic Annotation Pipeline (PGAAP) (http://www.ncbi.nlm.nih.gov/genome/annotation_prok). A total of 3,465 candidate protein-coding sequences (CDSs) were predicted (accounting for 93.85% of the total sequences), and 94 RNA genes were identified. The CDSs were searched against the KEGG and Clusters of Orthologous Groups (COG) databases to analyze the gene functions and metabolic pathways (8). In all, 2,549 CDSs were assigned to COG families and 1,521 CDSs were included in 100 pathways.

The range of substrate depends on the variety of metabolic pathways. Eighty-seven annotated genes were involved in the metabolism of aromatic compounds such as benzoate, bisphenol, fluorobenzoate, dioxin, xylene, toluene, polycyclic aromatic hydrocarbon, and ethylbenzene in strain YC-RL1. 11 Furthermore, 11 KEGG pathways related to aromatic degradation were annotated in YC-RL1. These data may explain the high degradation ability of strain YC-RL1 toward various aromatics. The genome information of strain YC-RL1 will provide further information for its practical application in the bioremediation process.

### Nucleotide sequence accession numbers. 

The complete genome sequence of *Arthrobacter* sp. YC-RL1 was deposited at DDBJ/EMBL/GenBank under the accession number LCYH00000000. The version described in this paper is the first version, LCYH01000000.
